# Supplementary material for: Visual adaptation and microhabitat choice in Lake Victoria cichlid fish
Source: R Soc Open Sci. 2019 Mar 27;6(3):181876. doi: 10.1098/rsos.181876 (PMC6458373; doi:10.1098/rsos.181876)
Supplement: Supplementary Methods and Results [file rsos181876supp1.docx]

## Electronic Supplementary Material

1. **Supplementary Methods:** fish groups used for preference trials (Table S1); normalized light conditions at Python Islands and in the laboratory (Figure S1); experimental tank with the two light environments (Figure S2); estimated proportion of incident light captured by the three main photopigments of *Pundamilia* (Figure S3).
2. **Supplementary Results:** fitted model for shallow light preference (Tables S2), preference for the rearing environment (Table S3) and preference strength (Table S4); correlations between fish activity and preference (Figure S5) and analysis in 15-minute blocks (Figure S6).

## Supplementary Methods

**Table S1:** Fish groups tested in this study, indicating species, rearing environment and age (''nn'' denotes *P. sp. ‘nyererei-like’* mother and father; ‘pp’ denotes *P. sp. ‘pundamilia-like’* mother and father; 'np' denotes *P. sp. ‘nyererei-like’* mother and *P. sp. ‘pundamilia-like’* father; 'pnpn' denotes 'pn' mother and 'pn' father; numbers indicate clutch numbers with different parents). Hybrid groups were tested twice; ‘pp’ and ‘nn’ groups were tested a third time (with the exception of three deep-reared ‘nn’ groups that were tested only twice (marked in grey).

| Group ID | Species | Rearing environment | Age (weeks) |
| --- | --- | --- | --- |
| nn24a | *P. sp. ‘nyererei-like’* | Shallow | 12 |
| nn24b | *P. sp. ‘nyererei-like’* | Shallow | 12 |
| nn25 | *P. sp. ‘nyererei-like’* | Shallow | 12 |
| nn27 | *P. sp. ‘nyererei-like’* | Shallow | 8 |
| nn28 | *P. sp. ‘nyererei-like’* | Shallow | 8 |
| nn23 | *P. sp. ‘nyererei-like’* | Deep | 12 |
| nn24a | *P. sp. ‘nyererei-like’* | Deep | 12 |
| nn24b | *P. sp. ‘nyererei-like’* | Deep | 12 |
| nn27 | *P. sp. ‘nyererei-like’* | Deep | 8 |
| nn28 | *P. sp. ‘nyererei-like’* | Deep | 8 |
| np6a | Hybrids | Shallow | 32 |
| np6b | Hybrids | Shallow | 32 |
| pnpn6a | Hybrids | Shallow | 24 |
| pnpn6b | Hybrids | Shallow | 24 |
| pnpn6c | Hybrids | Shallow | 24 |
| np6a | Hybrids | Deep | 32 |
| np6b | Hybrids | Deep | 32 |
| pnpn6a | Hybrids | Deep | 24 |
| pnpn6b | Hybrids | Deep | 24 |
| pnpn6c | Hybrids | Deep | 24 |
| pp13 | *P. sp. ‘pundamilia-like’* | Shallow | 28 |
| pp14a | *P. sp. ‘pundamilia-like’* | Shallow | 20 |
| pp14b | *P. sp. ‘pundamilia-like’* | Shallow | 20 |
| pp15a | *P. sp. ‘pundamilia-like’* | Shallow | 8 |
| pp15b | *P. sp. ‘pundamilia-like’* | Shallow | 8 |
| pp11 | *P. sp. ‘pundamilia-like’* | Deep | 36 |
| pp13 | *P. sp. ‘pundamilia-like’* | Deep | 28 |
| pp14 | *P. sp. ‘pundamilia-like’* | Deep | 20 |
| pp15a | *P. sp. ‘pundamilia-like’* | Deep | 8 |
| pp15b | *P. sp. ‘pundamilia-like’* | Deep | 8 |

**Figure S1:** Light conditions at Python Islands and in the laboratory - normalized by maximum irradiance in the field (at 583nm). Left panel: Downwelling irradiance in the natural habitats of *P. sp. 'pundamilia-like'* (0.5-2m depth; blue curve) and *P. sp. 'nyererei-like'* (0.5-5m depth, red curve). Right panel: Downwelling irradiance in the ‘shallow’ (blue curve) and ‘deep’ (red curve) light treatments in the laboratory. Curves represent averages of multiple measurement series; grey vertical lines indicate the maximum sensitivity of the three main photoreceptors of *Pundamilia.*

**Figure S2:** Estimated proportion of incident light captured by the three main photopigments of *Pundamilia*, for the natural light spectra (left panel) and the laboratory spectra (right panel). This was done by multiplying the irradiance curves with simplified spectral sensitivity curves of the *Pundamilia* photoreceptors (assuming Gaussian functions with the following peak sensitivities of the short-, middle- and long-wavelength-sensitive opsins: SWS 453nm, RH2 531nm, LWS 565nm; Carleton et al. 2005 Mol. Ecol. 14:4341–4353) and subsequent normalisation. Bars are averages of multiple spectrometry measurement series (as in Figure S1) with standard errors. Light blue bars represent the *P. sp. ‘pundamilia-like’* or shallow light condition, and orange bars represent the *P. sp. ‘nyererei-like’* or deep light condition. In both field and laboratory conditions, the ‘deep’ light condition generates lower SWS and higher LWS light capture than the ‘shallow’ light condition, with laboratory conditions slightly exaggerating the differences.


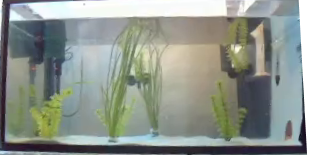

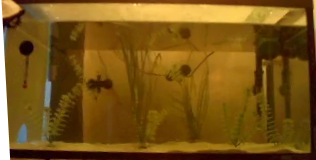


**Figure S3:** Experimental tank. In this photograph, the left side was set-up with the shallow-water-mimicking light condition and the right side with the deep-water-mimicking light condition.

1. **Supplementary Results**

**Table S2.** Summary of Satterthwaite's ANOVA analysis for shallow light preference, with F-statistics, degrees of freedom (numerator and denominator) and p-values.

Full starting model: *lmer (preference~species+rearing+activity+age+(family/group/repeat))*

| **Response variable** | **Main effects** | **F** | **Num df** | **Den df** | **p-value** |
| --- | --- | --- | --- | --- | --- |
| Preference for shallow light condition | Minimum adequate model |  |  |  |  |
|  | *Rearing environment* | 6.947 | 1 | 42.638 | 0.012 |
|  | *Activity* | 8.383 | 1 | 69.742 | 0.005 |
|  | Dropped terms |  |  |  |  |
|  | *Species* | 1.284 | 2 | 9.273 | 0.322 |
|  | *Age* | 2.830 | 1 | 31.248 | 0.103 |

**Table S3.** Summary of Satterthwaite's ANOVA analysis exploring the effect of ‘repeat’ on preference for the rearing light environment, for shallow-reared and deep-reared fish. Given are F-statistics, degrees of freedom (numerator and denominator) and p-values.

| **Response variable** | **Main effects** | **F** | **Num df** | **Den df** | **p-value** |
| --- | --- | --- | --- | --- | --- |
| Preference for rearing light condition | Deep-reared fish |  |  |  |  |
|  | *Repeat* | 7.382 | 1 | 23.618 | 0.012 |
|  | Shallow-reared fish |  |  |  |  |
|  | *Repeat* | 0.706 | 1 | 33.290 | 0.407 |

**Table S4.** Summary of Satterthwaite's ANOVA analysis for absolute light preference (i.e. preference strength; deviation from 0.5), with F-statistics, degrees of freedom (numerator and denominator) and p-values.

| **Response variable** | **Main effects** | **F** | **Num df** | **Den df** | **p-value** |
| --- | --- | --- | --- | --- | --- |
| Absolute preference | Minimum adequate model |  |  |  |  |
|  | *Activity* | 14.387 | 1 | 74 | <0.001 |
|  | Dropped terms |  |  |  |  |
|  | *Species* | 0.105 | 2 | 74 | 0.901 |
|  | *Repeat* | 0.197 | 1 | 74 | 0.659 |
|  | *Age* | 0.993 | 1 | 74 | 0.322 |
|  | *Rearing environment* | 1.403 | 1 | 74 | 0.240 |

**Figure S4:** Preference for the rearing light environment (blue bars – shallow-reared fish; yellow – deep-reared fish), with error bars indicating standard errors. Deep-reared fish decreased their preference for the rearing environment over subsequent repeats (F_1,23.618_=7.3824, p=0.01213) and as a result expressed a significantly weaker preference for the rearing environment overall, compared to shallow-reared fish (F_1,70.204_=22.419, p<0.001). Blue bars: shallow-reared fish; yellow bars: deep-reared fish. Numbers above bars indicate the number of test groups.

**Fish activity**

**Figure S5:** Scatter plot of fish activity (defined as the total number of crossings in a trial) with (a) preference for the shallow light condition (F_1, 69.742_=8.383, p=0.005) and (b) preference strength (irrespective of preferred environment; F_1, 71.837_=14.945, p=0.0002) in *P. sp. ‘pundamilia-like’* (blue), hybrids (purple) and *P. sp. ‘nyererei-like’* (red).


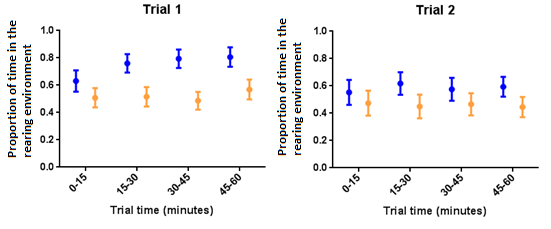


**Figure S6:** The effect of rearing environment in shallow-reared (blue) and deep-reared (yellow) fish was most pronounced in the first repeat and decreased with subsequent testing (see main text and Figure S4). To see whether preferences for the familiar environment already decreased during the first and second repeat, we calculated preferences in 4 blocks of 15 minutes. a) Preferences for the familiar environment did not decrease in the course of the first repeat. b) Also in repeat 2, preferences remained constant over the 60-minute trial duration, for both shallow-and deep-reared fish. Thus, we did not see signs of increasing familiarity with the novel environment during the repeats.
